# Should the surgeon or the general practitioner (GP) follow up patients after surgery for colon cancer? A randomized controlled trial protocol focusing on quality of life, cost-effectiveness and serious clinical events

**DOI:** 10.1186/1472-6963-8-137

**Published:** 2008-06-25

**Authors:** Knut M Augestad, Barthold Vonen, Ranveig Aspevik, Torunn Nestvold, Unni Ringberg, Roar Johnsen, Jan Norum, Rolv-Ole Lindsetmo

**Affiliations:** 1 Norwegian Centre for Telemedicine, Norway; 2 Department of Gastrointestinal Surgery, University Hospital of North Norway, Norway; 3 Institute of Clinical Medicine, Tromsø University, Norway; 4 Department of Surgery. Helgeland Hospital Trust, Mo i Rana, Norway; 5 Department of Surgery. Nordland Hospital Trust, Bodø, Norway; 6 Institute of Community Medicine, University Hospital of North Norway, Norway; 7 Northern Norway Regional Health Authority, Bodø, Norway; 8 Institute of Public Health and General Practice, Norwegian University of Science and Technology, Trondheim, Norway; 9 University Hospitals, Case Medical Centre, Cleveland, Ohio, USA

## Abstract

**Background:**

All patients who undergo surgery for colon cancer are followed up according to the guidelines of the Norwegian Gastrointestinal Cancer Group (NGICG). These guidelines state that the aims of follow-up after surgery are to perform quality assessment, provide support and improve survival. In Norway, most of these patients are followed up in a hospital setting. We describe a multi-centre randomized controlled trial to test whether these patients can be followed up by their general practitioner (GP) without altering quality of life, cost effectiveness and/or the incidence of serious clinical events.

**Methods and Design:**

Patients undergoing surgery for colon cancer with histological grade Dukes's Stage A, B or C and below 75 years of age are eligible for inclusion. They will be randomized after surgery to follow-up at the surgical outpatient clinic (control group) or follow-up by the district GP (intervention group). Both study arms comply with the national NGICG guidelines. The primary endpoints will be quality of life (QoL) (measured by the EORTC QLQ C-30 and the EQ-5D instruments), serious clinical events (SCEs), and costs. The follow-up period will be two years after surgery, and quality of life will be measured every three months. SCEs and costs will be estimated prospectively. The sample size was 170 patients.

**Discussion:**

There is an ongoing debate on the best method of follow-up for patients with CRC. Due to a wide range of follow-up programmes and paucity of randomized trials, it is impossible to draw conclusions about the best combination and frequency of clinic (or family practice) visits, blood tests, endoscopic procedures and radiological examinations that maximize the clinical outcome, quality of life and costs. Most studies on follow-up of CRC patients have been performed in a hospital outpatient setting. We hypothesize that postoperative follow-up of colon cancer patients (according to national guidelines) by GPs will not have any impact on patients' quality of life. Furthermore, we hypothesize that there will be no increase in SCEs and that the incremental cost-effectiveness ratio will improve.

**Trial registration:**

This trial has been registered at ClinicalTrials.gov. The trial registration number is: NCT00572143

## Background

An increasing incidence of colorectal cancer (CRC) has been observed in Norway during the past decades. According to the Cancer Registry of Norway, 2296 new cases of colon cancer were reported in 2006, making the disease among the most common type of cancer in both genders. The incidence in Norway is significantly higher than in the other Nordic countries [1]. The background for this discrepancy is unknown [2]. The overall five-year survival rate in 1993–97 was 56% for males and 60% for females [3]. At the time of diagnosis, about two thirds of patients had undergone resection, but 30–50% of them relapsed and died of the disease [4].

There is an ongoing debate on the best follow-up regimen for patients with CRC [5-7]. A meta-analysis published in 2002, including 1342 patients, demonstrated that intensive follow-up in CRC was associated with an absolute reduction in 5-year all-cause mortality of 10% [8]. Other trials have revealed similar results [9]. Due to the wide range of follow-up programmes employed, it is impossible to draw conclusions on the best combination and frequency of clinic visits, blood tests, endoscopic procedures, and radiological examinations that maximize the clinical outcome. Furthermore, the potential harms and costs of an intensified follow-up programme have not been clarified. The Norwegian Gastrointestinal Cancer Group (NGICG) revised its follow-up guidelines (Table 1) in 2007 [10]. According to these guidelines, most of the follow-up can be done by GPs. The aims of the follow-up programme are to perform quality assessment, provide support and improve survival. Most relapses (80%) of colon cancer are detected within the first three years of follow-up [11]. Based on this knowledge, the regular check-ups are recommended at three- month intervals for the first two years and then every six months. A similar CRC surveillance guideline was presented in 2005 by the American Society of Clinical Oncology (ASCO) [12].

**Table 1:** Follow-up of patients with colon cancer as proposed by the Norwegian Gastrointestinal Cancer Group (NGICG).

**Examination**	**Months postoperative**														
	
	1	3	6	9	12	15	18	21	24	30	36	42	48	54	60
Chest x-ray			X		X		X		X		X		X		X
Ultrasound liver			X		X		X		X		X		X		X
Coloscopy			X										X		
CEA measurement^a^	X	X	X	X	X	X	X	X	X	X	X	X	X	X	X
Clinical examination	X	X	X	X	X	X	X	X	X	X	X	X	X	X	X

This guideline is employed at Norwegian hospitals and will be used in both trial arms.

^a^Only patients with elevated CEA prior to surgery are requested to undergo this test during the follow-up.

During recent years, several critical articles have been published arguing against present follow-up procedures. So far, most studies addressing follow-up of CRC patients have been performed in a hospital setting. According to a recent Cochrane review, further research is needed, focusing on follow-up schedules in either a hospital setting or in general practice [6]. Whereas systematic postoperative surveillance has been extensively studied with regard to cure and survival, the possible benefits in terms of improved palliative care and/or quality of life have been less widely documented.

The purpose of this study is to obtain insight into quality of life among colon cancer patients followed up in a hospital outpatient setting or by their GPs and into the incremental cost-effectiveness ratio from the point of view of health care and of society.

## Methods and Design

### Study design

**Figure 1:**
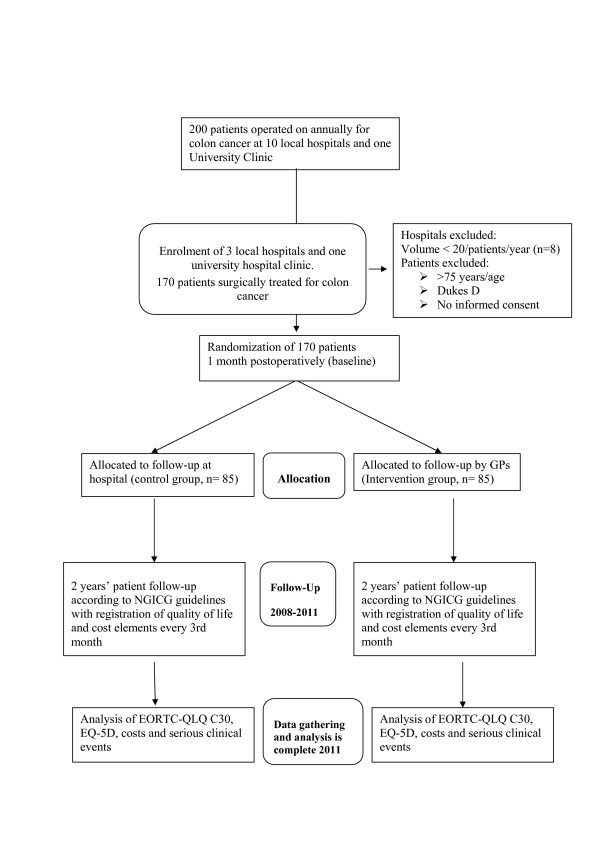
Trial Flow Chart

This trial is a multi-centre randomized controlled study where patients will either be randomized to follow-up at the hospital (control group) or by their GP (intervention group) (Figure 1).

### Participants

#### Patients

Patients are eligible if they undergo surgery for colon cancer with histological grade Dukes's Stage A, B or C. Patients receiving postsurgical adjuvant chemotherapy (Dukes C) are also eligible. We define a colon cancer as a tumour located 15 cm above the anal verge by coloscopy or a tumour at or above the level of the sacral promontory as seen at the time of surgery. Annually, approximately 200 patients with colon cancer undergo surgery in the catchment area of the North Norwegian Hospital Trust (a population of 470,000).

#### Hospitals

There are three hospital trusts and one university hospital (UNN HF) (in total 11 hospital units) located within the North-Norwegian Health Region. Three hospitals (located in the cities of Harstad, Mo, and Bodø) have an annual volume of more than 20 colon cancer procedures; the University Hospital operates on about 110 patients with suspected CRC cancer annually and approximately 60 of them have a colon cancer disclosed. The University Hospital and these three local hospitals consented to participate. All patients who undergo surgery for colon cancer are followed up at the surgical outpatient clinic at each hospital.

#### GPs

In North Norway, 550 GPs refer patients with suspected colon cancer to the local hospitals. They are situated in all communities in North Norway. All GPs were informed by mail about the purpose of the study and invited to participate. None refused to participate.

### Intervention

Patients are randomized to follow-up either by their GP (intervention) or at the surgical outpatient clinic. All patients will receive their first postoperative check-up at the hospital which performed the surgery (baseline, 1 month). A clinical examination will then be performed and information about the histology and the results of the surgery will be provided. If the patient gives informed consent, randomization is performed. Patients randomized to GP follow-up are referred to their GP. This referral contains information about the surgery and any complications, Dukes's staging, guidelines for follow-up and behavioural strategy in the case of a SCE.

#### Guidelines for follow-up

NGICG revised its guidelines in 2007 [10] (Table 1), and the GPs were provided with a guideline form based upon this recommendation. Most relapses of colon cancer are detected within the first three years of follow-up. On the basis of this knowledge, the regular check-ups are performed at three-month intervals for the first two years and then every six months.

All patients with elevated CEA prior to surgery are requested to undergo this test at every postoperative clinical examination. Chest x-ray and ultrasound are performed on a regular basis. Colonoscopy is performed twice during the follow-up period. The follow-up guideline is similar in both arms (Table 1).

#### When recurrence is suspected

All GPs are given written instructions on what to do if recurrence is suspected. A set of serious clinical events (SCEs) is defined as: Colonoscopy verifies recurrence of CRC. Elevated levels of CEA are shown by repeated measurements. Blood in stool is detected by the Hemofec test. Unexplained abdominal pain. Unexplained weight loss of 5 kg during the last three months. Cancer-suspect lesions detected by rectal examination. Lymphandenopathy. Metastasis-suspect lesions shown by chest x-ray or ultrasound of liver. If an SCE occurs, the patient must be referred promptly to hospital.

#### Information to the patient

All patients included in the study are given a folder explaining the purpose of the study. Follow-up guidelines are provided with detailed information concerning the clinical examinations, CEA measurements, x-ray/ultrasound procedures and colonoscopy.

#### QOL questionnaires

The EQ-5D and EORTC-QLQ C30 questionnaires for the two-year follow-up period (at 1, 3, 6, 9, 12, 15, 18, 21, 24 month follow-up) are given to the patient at baseline examination.

Economic evaluation questionnaire: A questionnaire has been developed to enable calculation of costs related to patient examinations. This questionnaire is incorporated in the QOL questionnaire, and patients are requested to fill out the questionnaire at 1, 3 6, 9, 12, 15, 18, 21 and 24 months postoperatively. The cost elements will include patient- and family-related costs due to outpatient visits, GP visits, laboratory tests, radiographs/ultrasound, examinations due to suspected relapse, treatment of relapse, travelling, production losses, co-payments and other patient expenses.

### Control arm

Patients in the control group consist of patients randomized to regular follow-up at the hospital's surgical outpatient clinic. All these patients are followed up according to the NGICG guidelines. This follow-up is performed by consultants or internship doctors in digestive surgery.

### Objectives

#### Primary objective

We aim to compare quality of life and costs of follow-up among patients followed up by their local GP or at the surgical outpatient clinic. We hypothesize that patients followed up by their GP will experience similar or higher scores on quality of life measures and lower costs during follow-up.

#### Secondary objective

We aim to test whether the incidence of SCEs will be similar for patients followed up by their GP and patients in the control group. We hypothesize that patients followed up by their GP will not have an increase in time to detection or in the frequency of SCEs.

### Outcome measures

#### Primary outcome (measured at 1, 3, 6,9,12,15,18,21 and 24 months)

#### Quality of life

EQ-5D is a standardized generic instrument for use as a measure of health outcome. Applicable to a wide range of health conditions and treatments, it provides a simple descriptive profile and a single index value for health status. EQ-5D measures five dimensions of health-related quality of life (HRQOL): mobility, self-care, usual activities, pain/discomfort and anxiety/depression. EQ Visual Analogue Scale: The EQ VAS records the respondent's self-rated health status on a vertically graduated (0–100) visual analogue scale [13]. Quality of life assessment based upon the European Organization for Research and Treatment of Cancer Quality of Life questionnaire (EORTC QLQ C-30); EORTC QLQ C-30 incorporates nine multi-item scales: five functional scales (physical, role, cognitive, emotional and social); three symptom scales (fatigue, pain, nausea/vomiting); and a global health status/QOL scale. Six single-item scales are also included (dyspnoea, insomnia, appetite loss, constipation, diarrhoea and financial difficulties) [14].

#### Economic analysis

A cost-effectiveness analysis will be performed alongside the randomized controlled trial. A Markov model will be employed. If the trial reveals no difference in health outcomes or quality of life of the patients, a cost-minimization analysis will be carried out. If we reveal a difference in quality of life between the two groups, we will implement a cost-utility analysis. The economic evaluation will have a health care and societal perspective. Resources used in both treatment arms will be registered prospectively based on reports by the patients and on the hospital patient records. Costs related to consultations at the primary and secondary care level, both at baseline and up to 24 months after randomization, including direct medical costs, indirect costs (production losses), travel costs and patient-/family-related costs will be registered. Quality of life measurements will be collected both at baseline and at several time points during the intervention. A 3% discount rate will be used to discount future costs and benefits. The cost elements will include costs related to outpatient visits, GP visits, laboratory tests, radiographs/ultrasound, examinations due to suspected relapse, treatment of relapse, travelling/transportation, production losses, co-payments and other patient/family expenses. The net costs and outcomes of the two treatment options will be compared and presented as incremental cost-effectiveness ratios.

### Secondary outcome measures

#### Serious clinical events (SCEs)

Time to detection of recurrences and serious clinical events (SCEs) defined as: Colonoscopy-verified recurrence of cancer disease. Increase in CEA measurements shown by repeated measurements. Blood in stool detected by the Hemofec test. Unexplained abdominal pain. Unexplained weight loss of 5 kg during the last three months. Cancer-suspect lesions detected in rectal examination. Lymphandenopathy. Metastatic suspect lesions shown by chest x-ray or ultrasound of liver.

### Sample size

Sample size calculations are according to Campbell's guidelines [15]

Alpha and beta were set at 0.05 and 0.2 respectively and tests were two-sided. We hypothesize that patients followed up by their GP will have a minor to moderate increase in the Global QoL scores (EORTOC-QLQ C30). This moderate effect size equals an increase of 10 units on the Global QOL [16]. Patients with colon cancer (local disease) show a standard deviation of approximately 20 (global QOL) [16]. Allowing for a dropout rate of 20%, to detect this difference with confidence we required a total sample size of 170 patients. Sample size calculations based upon EQ-5D showed similar results [17].

### Randomization

Randomization will be done at the 1-month check-up (baseline) after the patients have given informed consent. The randomization service is web-based and managed by the Norwegian University of Science and Technology. Patients are stratified according to the Dukes's staging and whether they have a stoma. Patients allocated to general practice follow-up may be referred back to surgical clinics at any point in the study; similarly, patients in the surgeon-led follow-up group are free to consult their GP at any time during the study.

### Data gathering

Data are being collected for patients in the intervention and the control group in identical ways. QoL and "cost questionnaires" are sent every three months (after each follow-up appointment) by the patients to the trial centre up to 24 months postoperatively. These questionnaires are optically readable, data being consecutively collected in a trial database.

SCEs are registered via hospital chart review at 24 months postoperatively. This follow-up period will allow identification of most SCEs, since tumour recurrence typically occurs within two years of surgery [11].

The study has been approved by the Norwegian Data Inspectorate. All data will be handled with strict confidentiality, and study reports or presentations will maintain the anonymity of patients, surgeons, GPs and hospitals. Data collection will be complete by the end of 2010.

### Analysis

We will use the intention-to-treat principle when analysing data. Furthermore, we will employ Fayers guidelines to handle missing data [18]. Treatment arms will be compared with respect to potential covariates using continuous and categorical univariable analyses. These will include variables relating to patients (age, sex, comorbidities, cost), treatment (surgical resection, surgical complications, adjuvant therapy), tumour (Dukes's Stage), hospital surgical outpatient clinic (cost, patient satisfaction, QoL), GP practice (cost, patient satisfaction, QoL). The cost elements will include costs related to outpatient visits, GP visits, laboratory tests, radiographs/ultrasound, and examinations due to suspected relapse, treatment of relapse, travelling/transportation, production losses, co-payments and other patient expenses. The net costs and outcomes of the two treatment options will be compared and presented as cost-effectiveness ratios. If the trial reveals no difference in health outcomes or quality of life for the patients, a cost-minimization analysis will be carried out. If we find a difference in quality of life or overall survival rates between the two groups, we will use a cost-utility approach. The economic evaluation will have a societal and health care perspective.

The method of analysis, including adjusting for covariates will comply with the CONSORT statement [19]. The results will be expressed as odds ratios for binary outcomes, hazard ratios for time- to-event outcomes or mean differences for continuous outcomes with corresponding standard errors, 95% confidence intervals, and associated p-values. P-values will be reported to three decimal places with p-values less than 0.001 reported as p < 0.001. For all tests we will use alpha = 0.05 level of significance.

## Ethics

The Regional Committee for Medical Research Ethics, North Norway approved this protocol (P REK NORD 79/2006). Patients must provide written consent before entering the trial.

## Discussion

Whereas systematic postoperative surveillance has been extensively studied with regard to cure and survival, the possible benefit of surveillance with regard to better outcome of palliative care and/or quality of life has been less widely documented. It has been shown that follow-up programmes can lead to psychological stress [20]. Kjeldsen concluded that patients receiving frequent follow-ups had greater confidence in the check-ups, but the improvement in the health-related quality of life was only marginally improved. The study concludes that the minor improvement in health-related quality of life does not justify an expensive and frequent follow-up schedule [21]. Kievit published a meta-analysis in 2002 arguing for an increased focus on quality assessment and patient support in a follow-up programme, as improved survival is realized in only a few patients [22]. According to this study, there is no need for routine follow-up to be performed by a surgeon. Patients with asymptomatic but incurable disease (9% in Korners study) [23], raise serious ethical concerns. Even with today's chemotherapy regimens with significant effects in terms of response rate and overall survival, cure is rarely seen when patients are beyond salvage curative surgery [24]. A meta-analysis of six randomized trials demonstrated that intensive follow-up in colorectal cancer was associated with an absolute reduction in all-cause 5-year mortality of 10%; however, only two percent was attributable to cure from salvage re-operations. Renehan et al postulate that other factors, such as increased psychological well-being and/or altered lifestyle, and/or improved treatment of coincidental disease may contribute to the remaining lives saved [25].

The geography of Norway makes the costs of travelling a significant burden. In North Norway in particular, the population is scattered throughout a large geographic area, making the cost of travel to a specialist examination considerable. The specialists are mainly located in a few main cities. New regulations requiring each hospital to cover travel expenses have resulted in a stronger focus on these costs in recent years. At present, 5% of the total budget of the Regional Health Authority of North Norway is spent on travelling/transportation. The cost of travelling was earlier funded by the Norwegian Insurance Administration. If a follow-up programme (i.e. clinical examination and medical history) can be run by the patient's GP, there are obvious reasons to believe that the total costs of such a programme could be reduced.

Whereas there is evidence of survival gain related to intensive follow-up programmes, the burden of cost to the health care service is considerable [26-28]. Renehan et al. published a study in 2004 arguing that intensive follow-up after curative resection for colorectal cancer is economically justified and should be standard practice [29]. However, a Danish study concludes the opposite, stating that follow-up after colorectal cancer surgery is not cost-effective compared to several other procedures, including screening for CRC [21]. The cost-effectiveness of the Norwegian guidelines was documented cost-effective in a prior study when applied in a model-based study implementing data from the literature. The basic cost of the NGICG recommended programme was GBP£ 1,232 per patient. Including extended investigation due to suspected relapse in 45% of cases, the figure rose to £ 1,943 per patient [30]. However, Korner et al [23] argue against this study. The total programme cost in this study was Euro € 228,117 (US 280,994 dollars), translating into € 20,530 (US 25,289 dollars) for one surviving patient after salvage surgery. This cost is less than the one calculated by Norum, but the costs are calculated at different times and in different settings, making a direct comparison useless. Costs are hard to compare with publications from other countries because of different reimbursement policies. Finally, they [23] argue whether the continuing implementation of such a program and costs are justified should be further debated.

The setting of follow-up may have an impact on patients' well-being and satisfaction with care. A recently published study showed that there were no differences in score for quality of life among patients with colon cancer, randomized to follow-up by GPs or specialists [31]. However, this study is based upon follow-up procedures that differ from those used in Norway (e.g. no regular radiological procedures), and there is no cost-effectiveness analysis. To our knowledge this is the first study addressing this problem. According to a Cochrane review, further research is needed [7]. Studies addressing follow-up of other cancer types by GPs have been performed, indicating for example that breast cancer patients can be safely followed up by their family physician without any concerns related to clinical outcome or health-related quality of life [32]. Recent studies have indicated that the GP has a place in the follow-up of many patients with cancer, also in the initial phase after treatment [33]. Patients trust their GP to provide competent care, especially when they have more complex health care needs on top of their cancer [34-36].

## Competing interests

The authors declare that they have no competing interests.

## Authors' contributions

The study was initiated by KMA and R–OL. KMA is the grant holder. All authors participated in the study concept and design. All authors reviewed and approved the final version of this manuscript.

## Pre-publication history

The pre-publication history for this paper can be accessed here:


